# Hybrid Effect of Steel Bars and PAN Textile Reinforcement on Ductility of One-Way Slab Subjected to Bending

**DOI:** 10.3390/molecules27165208

**Published:** 2022-08-15

**Authors:** Omar H. Hussein, Amer M. Ibrahim, Suhad M. Abd, Hadee Mohammed Najm, Saba Shamim, Mohanad Muayad Sabri Sabri

**Affiliations:** 1 Department of Civil Engineering, College of Engineering, University of Diyala, Baqubah 32001, Iraq; 2 Department of Civil Engineering, Zakir Husain College of Engineering and Technology, F/O Engineering and Technology, Aligarh Muslim University, Aligarh 202002, India; 3 Peter the Great St. Petersburg Polytechnic University, St. Petersburg 195251, Russia

**Keywords:** textile reinforced concrete (TRC), one-way slab, ductility, energy-based method

## Abstract

Textile reinforced concrete (TRC) has gained attention from the construction industry due to several characteristics such as its lightweight, high tensile strength, design flexibility, corrosion resistance and remarkably long service life. Some structural applications that utilize TRC components include precast panels, structural repairs, waterproofing elements and façades. TRC is produced by incorporating textile fabrics into thin cementitious concrete panels. However, in order to use this strengthening method in construction practice, a design model is required. Investigating the combined effect of conventional steel and textile reinforcement on the ductility behavior of composite TRC/RC one-way slab is vitally important. Therefore, the current study describes the proper methods of calculating the ductility of the composite concrete reinforced by a direct combination of conventional steel and textile reinforcement. Four methods are presented to calculate the ductility of the three considered one-way slab specimens. The three slabs having dimensions 1500 mm × 500 mm × 50 mm were reinforced by steel bars (SRC), by steel with one layer of carbon fabric (SRC + 1T), and by steel with two layers of carbon fabric (SRC + 2T). The three slab specimens were cast by the hand lay-up method, removed from the molds, cured, and then tested in flexure after 28 days using the four-point bending method. The obtained results and calculations revealed the non-reasonability of using the conventional method based on yielding of steel reinforcement as the only criterion in the ductility determination. The results also confirmed the suitability of using the energy-based method over other discussed methods in the calculation of the ductility for the hybrid reinforced members.

## 1. Introduction

Textile reinforced concrete (TRC) is a novel composite material prepared by incorporating continuous textile fabric into fine-grained concrete that consists of a cement binder and fine aggregates. The textile reinforcement materials consist of continuous-length yarns, which consist of many hundreds, thousands, or ten thousand filaments. The filaments in the yarn are divided into external (sleeve) filaments and internal (core) filaments. The sleeve filaments are only in full contact with the surrounding matrix while the core filaments are in less contact with the matrix; therefore, the tensile stresses are transferred from the matrix to the yarn sleeve filaments by adhesion, while the core filaments, which are in less contact with the matrix, work on friction. The TRC materials are quite suited for applications where strain hardening is desired, and where the steel reinforcement can be eliminated or can be used for structures that are subjected to seismic loads which require high ductility. Moreover, its economical aspect and exceptional bonding with concrete also promoted the use of the TRC strengthening technique.

Ductility refers to the ability of a material to sustain a plastic deformation before fracture. It is the most important characteristic of a structure, as it must be gained and improved in the structural members. The appropriate ductile behavior must be realized under the conditions of the ultimate load so that the ductility could be considered a favorable structural characteristic. However, the internal force redistribution and plastic hinge formation in statically indeterminate structures are related to ductility. Furthermore, the ductility gives a prior indication about the failure before its occurrence thus; the building can be evacuated [1].

The composite reinforced concrete (RC) members consist of brittle (concrete) and ductile (steel) materials. The mode of failure of an RC member is related to the amount of steel reinforcement. A balanced reinforcement ratio separates the ductile tensile failure from the sudden compression failure. The plastic deformation of the RC member occurs after the yielding of steel, and the deformation is increased without any loss in the load-carrying capacity at this stage. On the other hand, the inherent corrosive nature of steel along with its high weight-to-strength ratio promoted the investigation of several alternative reinforcement types such as fiber reinforced polymer (FRP) bars and textile reinforcement. Textile reinforcement such as carbon, AR glass, and Kevlar are mostly preferred for reinforcing concrete members as compared with the other reinforcement materials due to their excellent properties in terms of high tensile strength and modulus of elasticity [2]. The ductility of the elements reinforced using FRP have been discussed by a few researchers in the past [3,4]. The analysis and determination of the ductility characteristics of such elements still lack clear acceptance despite the several attempts achieved to realize this issue [1].

Textile reinforced concrete (TRC) has been successfully used in strengthening RC structural members such as beams and slabs [5,6,7,8,9]. The use of the TRC in strengthened concrete offers great advantages over FRP, such as high resistance to elevated temperature, non-toxicity, and enhanced compatibility with the structural member [10,11,12,13]. The TRC-strengthened layer provides a clear and cooperative contribution in bearing the stresses [14,15,16]. The TRC/RC composite slabs represent a suitable alternative to the conventional reinforced concrete slabs [17]. Yin et al. [18] studied the use of TRC as a tension cover in RC beams. It was concluded that the concrete flexural members reinforced by the textile and steel bar combination efficiently controlled the crack width extension. Additionally, it realized an improvement in serviceability and load-bearing capacity [18]. Papanicolaou and Papantoniou [15] and Yin et al. [18] calculated the ductility of the composite RC beam with a TRC tension cover according to the deformation-based method, which is concerned with the yield of the steel reinforcement. It was found that the ductility values were reduced for the composite (RC with TRC cover) beams as compared to the control or reference beam, thereby confirming the fact that the TRC tension cover has a significant contribution to the flexural capacity of the reinforced section. Consequently, the yielding load of the steel reinforcement was found to be enhanced, accompanied by an increase in deflection. It further led to a decrease in the ratio between the ultimate and yield deflection.

Generally, the FRP and textile reinforcement exhibit linear elastic behavior. Therefore, the ductility of the flexural concrete members that are reinforced by such materials cannot be determined using the deformation method since these materials do not present a yield. El Zareef and El Madawy [19] stated that the conventional ductility definitions are improper for calculating the ductility of the concrete beams reinforced by the FRP bars due to the lack of yield point. Accordingly, the ductility of the beams reinforced with the FRP bars was calculated using two different methods, namely the deformability-based method proposed by Jaeger et al. [20] & Zhi et al. [21] and the energy-based method suggested by Namaan and Jeong [4]. Abd and Ghalib [22] also calculated the ductility of two beams (1500 × 200 × 250 mm) reinforced by GFRP using the last two methods. However, the ductility values obtained by the two methods showed an obvious variance, i.e., the deformability method yielded the higher values as compared with the energy-based method. This can be attributed to the small difference in the slope of the load–deflection curve between the pre- and post-cracking stage. Thus, the elastic energy showed a considerable portion of the total energy. Furthermore, the addition of the FRP bars to the conventional steel reinforcement led to an improved ductility, although on a small scale, while the replacement of the steel reinforcement by the FRP bars resulted in the reduction in ductility due to the linear elastic behavior. Yuan and Chen [23] stated that the hybrid FRP–steel reinforcement caused higher extreme deformability and deflection, while the ductility did not necessarily improve. They reported that the hybrid FRP–steel reinforced beams exhibited a steep moment–curvature curve in the inelastic region, in contrast with the steel reinforced beams which showed a flat curve during this stage. Thus, the ultimate deflection and the plastic hinge length were increased in the case of hybrid beams. Foraboschi [24,25] stated that the beams reinforced with hybrid bars do not dissipate as compared with the beams reinforced by steel bars. The textile reinforcement materials are similar to FRP in that they yield linear elastic behavior up to their failure. Therefore, the ductility of the TRC members cannot be determined based on the conventional ductility factors.

This study aims to estimate the ductility of the TRC/RC composite one-way slabs with the use of four different methods. A comparison among them is also performed in order to determine the proper method for calculating the ductility. The three RC one-way slabs were the reference slab (SRC), the RC slab with one layer of carbon fabric (SRC + 1T), and the RC slab with two layers of carbon fabric (SRC + 2T); these were considered and fabricated using the hand lay-up method in wooden molds. The flexural behavior of the slabs was determined using the four-point method after 28 days of curing.

It has been observed in the literature that there are many existing experimental works that evaluate the ductility of the One-Way Slab [26,27,28,29,30,31,32,33,34,35]. However, there is no available experimental work evaluating the effect of the combined utilization of conventional steel and textile reinforcement on the ductility behavior of composite TRC/RC one-way slab, which is important to investigate. Moreover, research investigating the use of PAN (Polyacrylonitrile) as an alternative material for textile reinforcement is not addressed in the literature. In the present study, the PAN textile reinforcement was placed in contact with the steel reinforcement in order to investigate the mutual contribution of both the textile and steel reinforcement in bearing the stresses, which is the main novelty of this work. This information can be used as a reference for future studies and provide extensive data for studies on textile reinforced concrete.

## 2. Experimental Program

### 2.1. Concrete Matrix

**Figure 1:**
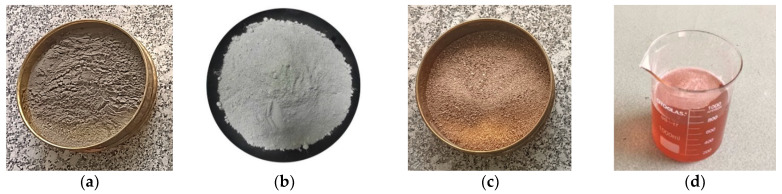
(**a**) OPC. (**b**) silica fume (SF). (**c**) fine aggregate. (**d**) superplasticizer.

The cement matrix used in this study consists of ordinary Portland cement, fine sand (150–600 µm), silica fume (micro-silica), and superplasticizer (hydrous solution of modified polycarboxylate) meeting the requirements of (ASTM-C-494) [36], as shown in Figure 1. Likewise, the matrix constituents and their quantities are summarized in Table 1.

### 2.2. Reinforcement Techniques

Two types of reinforcement are used in this study for fabricating the three samples of one-way slab, as discussed in the subsequent section.

#### 2.2.1. Conventional Steel Reinforcements

Both the main and secondary steel-reinforced bars were deformed and hot-rolled with a diameter of 4.45 mm. The tensile test of the steel reinforcement was carried out as per (ASTM A615/A 615M) [37]. The specimens were tested in the material laboratory of the Civil Engineering Department at Diyala University in Iraq. The results are summarized in Table 2.

#### 2.2.2. Textile Reinforcement Fabrics

**Figure 2:**
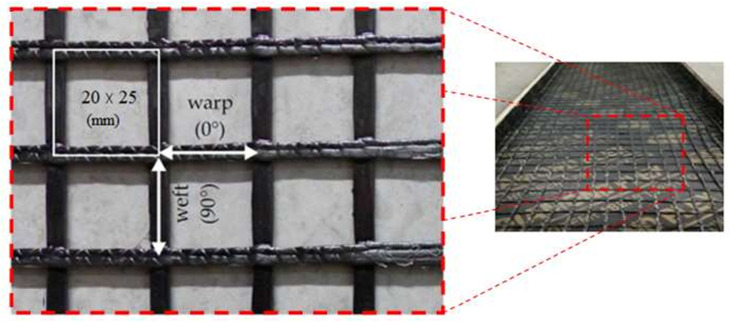
PAN (Polyacrylonitrile) textile.

**Figure 3:**
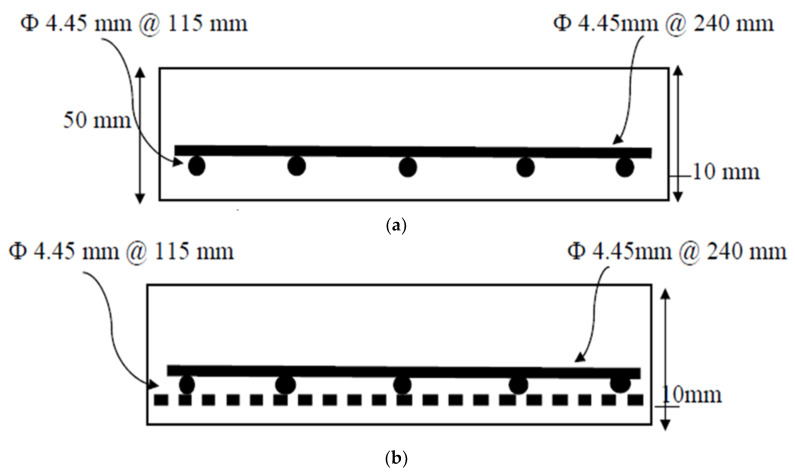

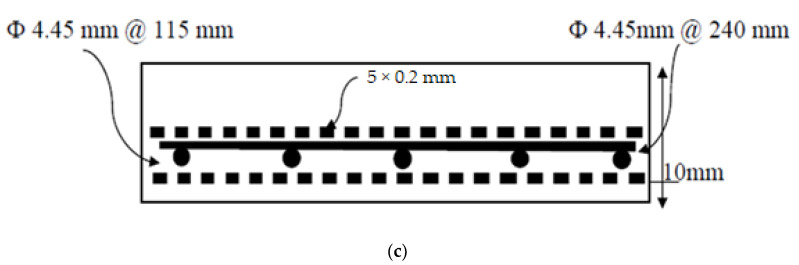
Reinforcement details for the three tested slabs. (**a**) SRC reinforcement details. (**b**) SRC + 1T reinforcement details. (**c**) SRC + 2T reinforcement details.

An innovative PAN (Polyacrylonitrile) textile reinforcement grid is used in this study, which has a grid size of 25 × 30 mm and a 12-k filament. This grid was supplied by Jiaxing Newtex Composites. Table 3 and Figure 2 show the manufacturer data sheet. Table 4 indicates the reinforcement details for the three slab specimens; and Figure 3 illustrates the reinforcement details for the three slab specimens.

### 2.3. One-Way Slab Preparation

#### 2.3.1. Mixing Procedures

A horizontal rotary mixer with the capacity of 0.06 m^3^, available in the structural laboratory of the Civil engineering department, College of Engineering, Diyala University was used to prepare the concrete mix. The mixing was as follows:

1. The required amounts of both dry cement and silica fume were mixed for 5 min to ensure silica fume dispersion between the cement particles.
2. The process of fine sand addition to the mixer continued for another 5 min.
3. A third of the total quantity of water was added, and the mixing continued for next 1–2 min.
4. The mixture of 2/3 residual water and superplasticizer was added to the rotary mixer, with the operation continued for further 4 min to assure that all components were mixed. Then, the mixing was continued manually when the rotary mixer was stopped, especially for the parts not reached by the mixer blades. After that, the rotary was operated to achieve sensible fluidity.

#### 2.3.2. Concrete Properties

Compressive tests, Tensile tests, Flexural tests, and Modulus of Elasticity were conducted as per (ASTM Designation C39/C39M–15a [38]), (ASTM Designation C496/C496M–11 [39]), (ASTM Designation C78/C78M–15a, [40]), and (ASTM Designation C469/C469M–14, [41]), respectively, on samples cast from each batch of the concrete one-way slab. Table 5 outlines the results of the tests conducted during the slab testing to assess the actual strength of the slab concrete mix.

#### 2.3.3. Casting and Curing Method Adopted

**Figure 4:**
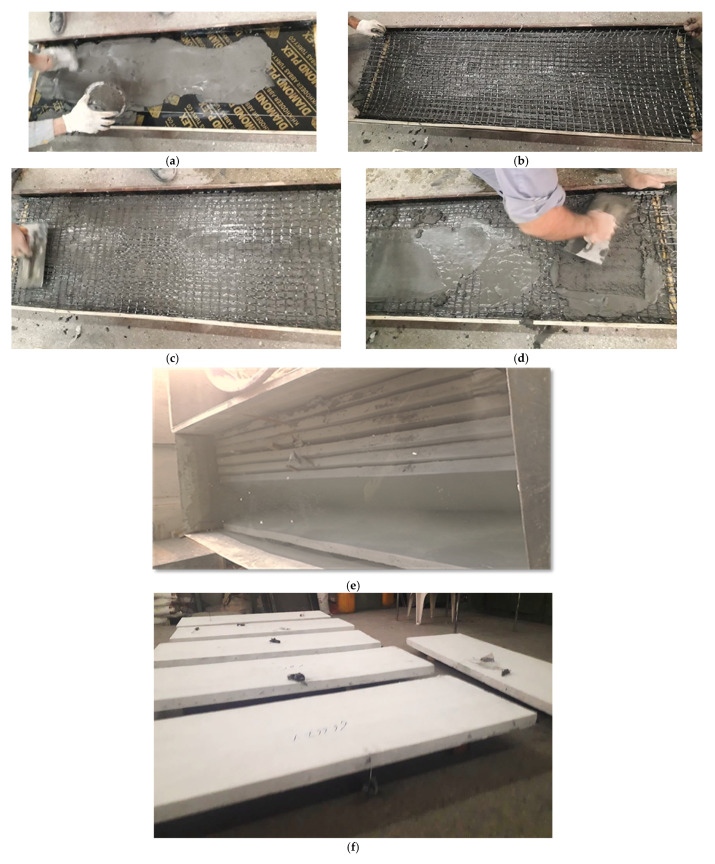
The slab specimens during the casting, curing, and painting processes. (**a**) Casting of concrete cover. (**b**) Placing of textile layer. (**c**) Pressing the textile layer (**d**) Top concrete casting. (**e**) Curing of slabs by immersing in water tank. (**f**) Finished view of slabs after painting.

Plywood molds with dimensions of 1500 *×* 500 *×* 50 mm were used for the casting of the three slab specimens. Firstly, the molds were oiled in order to inhibit the adhesion of the concrete onto the molds. Then, a 10 mm thick concrete layer was poured, above which the first layer of the PAN grid was placed, followed by the steel reinforcement grid. In the case of the SRC + 1T slab, the concrete was poured up to the top of the mold beyond the steel reinforcement grid. In the case of SRC + 2T, the second PAN layer was placed directly above the steel reinforcement grid, and then the concrete was poured onto the top of the mold. In the case of the reference slab, SRC, only the steel reinforcement grid was placed above the 10 mm thick concrete layer. Figure 4a–d shows the casting processes.

Curing is a process of fully controlling the hydration of the Portland cement in the concrete. It usually entails regulating the loss of moisture, and in certain cases, temperature [29]. Shade concrete work, covering concrete surfaces with hessian or gunny bags, spraying water, ponding technique, immersion in water, membrane curing, and steam curing are all several examples of curing methods.

For this project, the curing method adopted was total immersion in water (Figure 4e), where the samples were fully immersed in a water tank. It is a suitable method satisfying all the requirements of curing specifically, hydration promotion, shrinkage elimination and absorption. The slab specimens were removed from curing tank, dried, painted (Figure 4f), and then fixed with the strain gauges on their surfaces in order to test them in flexure after 28 days.

### 2.4. Instrumentation and Measurements

#### 2.4.1. Four-Point Bending Test

**Figure 5:**
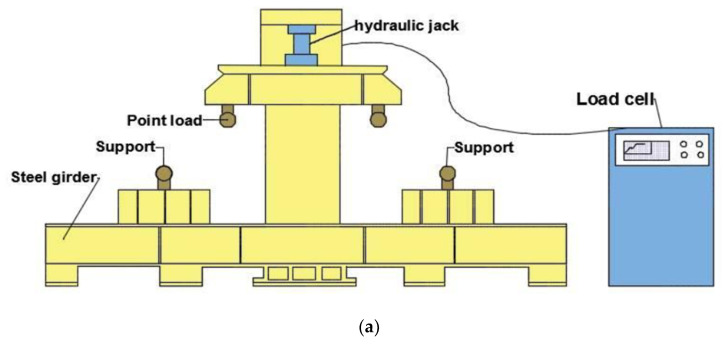

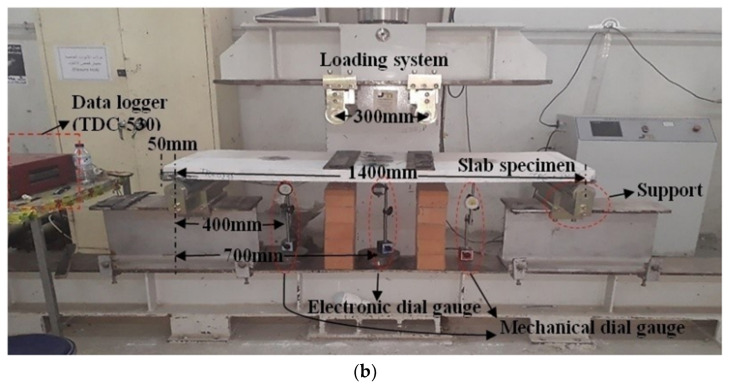
Four-point bending test set-up. (**a**) Schematic diagram of the testing machine. (**b**) Four-point bending test in laboratory.

The flexural test is considered as the primary test for evaluating the bending capacity and ductility of the TRC-RC one-way slab. The four-point bending test was executed to investigate the bending capacity of the three slab specimens. This was performed on a hydraulic Universal Testing Machine, with a maximum range capacity of 600 kN. The slabs were tested after 28 days. They were prepared, cleaned, and coated with white paint in order to reveal the possible cracks during testing. The slabs were placed, simply supported, over a clear span of 1400 mm and were tested under two concentrated loads applied at 550 mm from each support. The load was applied, and the corresponding strains, deflections, and crack widths were recorded for each 0.1–1 kN increment in load. The test was conducted in the structural laboratory of the Civil Engineering Department of the College of Engineering in Diyala University. Figure 5a,b illustrates the four-point bending test set-up.

#### 2.4.2. Deflection Measurement Devices

**Figure 6:**
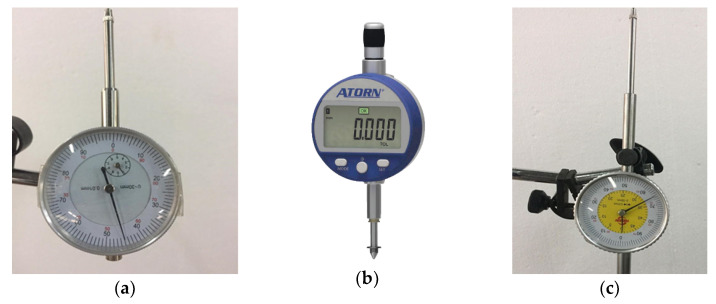
Dial gauges used in the present study. (**a**) Mechanical dial gauge. (**b**) Electronic dial gauge. (**c**) Mechanical dial gauge.

Three dial gauges were used to measure the vertical deflection (displacement) of the tested slab specimens; one electronic dial gauge was placed below the mid-span of the slab, while the other two mechanical dial gauges were placed at a distance of 300 mm from each support. The accuracy of all the three dial gauges was 0.01 mm. Figure 6a–c show the dial gauge used in the study. The deflection values obtained from the three gauges were directly recorded for each applied load.

#### 2.4.3. Strain Measurement Device

**Figure 7:**
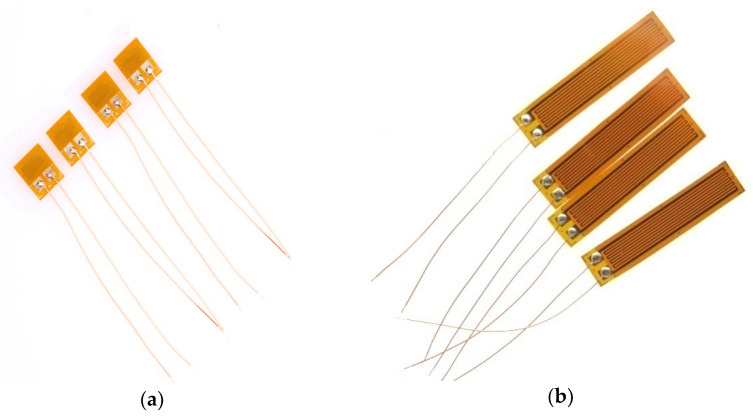
Types of strain gauges. (**a**) BF350-1AA. (**b**) BF120-20AA.

Strains were measured to determine the behavior of the composite one-way slab specimens against the applied stresses. The use of a highly accurate device is required to calculate the amount of strain in the concrete. The BF120-20AA and BF120-20AA strain gauges (see Figure 7a,b) produced by SNC Company were used to measure the compressive strain in concrete and the tensile strain in steel during the test. Table 6 indicates the properties of these strain gauges provided by the manufacturer.

## 3. Results and Discussion

### 3.1. Load Capacity

Table 7 demonstrates the test results for the three tested slab specimens. The results confirm that the combination of the steel reinforcement with one and two layers of PAN grid increased the load capacity. At yielding, the percentage of load enhancement was found to be 115% and 91.5% for SRC + 1T and SCR + 2T, respectively. Furthermore, the mid-span deflection was significantly increased at yield by about 120% in SRC + 1T and by 26% in SRC + 2T. An increment in the mid-span deflection at yield point (for SRC + 1T and SRC + 2T) may be accounted for the higher post cracking stiffness compared to SRC. The ultimate mid-span deflection was quite improved for both SRC + 1T and SRC + 2T by 77.8% and 85.4%, respectively, as compared with the SRC one-way slab. However, this improvement in the mid-span deflection is attributed to the slippage of the core filaments, which usually occurs at the last stage of loading.

These results of the present study are in agreement with the results of Papanicolaou et al. [15], except for one difference, which is the reduction in the mid-span deflection at the ultimate load. However, this difference is slight, but it can be attributed to the 19 mm mortar layer that separates the steel and textile reinforcement since this mortar is sufficient to enhance the bond and inhibit the slipping of the core filaments.

### 3.2. Load–Deflection Behavior

**Figure 8:**
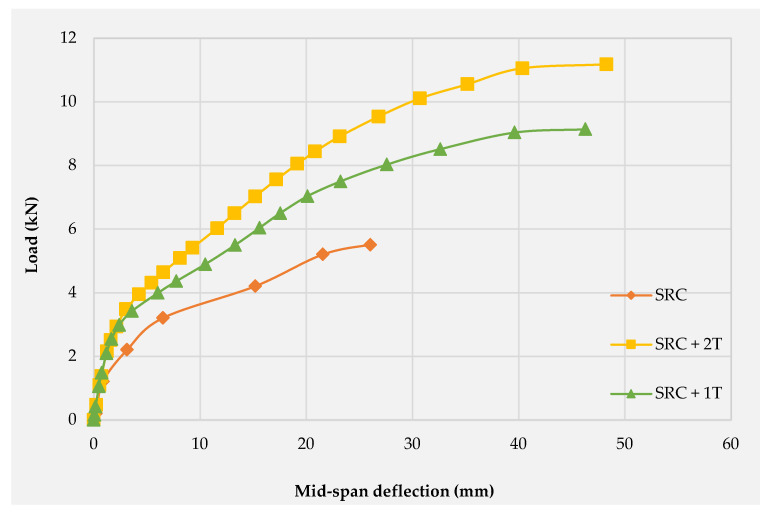
Load versus mid-span deflection curve for the three tested slabs.

Figure 8 shows the load–deflection curves for the three tested slabs. As can be observed, the two slabs (SRC + 1T and SRC + 2T) yieldeda higher cracking load with lower deflection as compared with the SRC slab; thus, the slope of the load–deflection curves are significantly increased for the two tested slabs during the uncracked linear elastic stage. The post-cracking region also showed higher slopes for the SRC + 1T and SRC + 2T curves as compared with the SRC curve. This is attributed to the improvement in the flexural stiffness realized by textile reinforcement. A more or less similar Load–Deflection Behavior for the textile-reinforced concrete was reported by Kim et al. [35].

### 3.3. Load–Strain Curve

**Figure 9:**
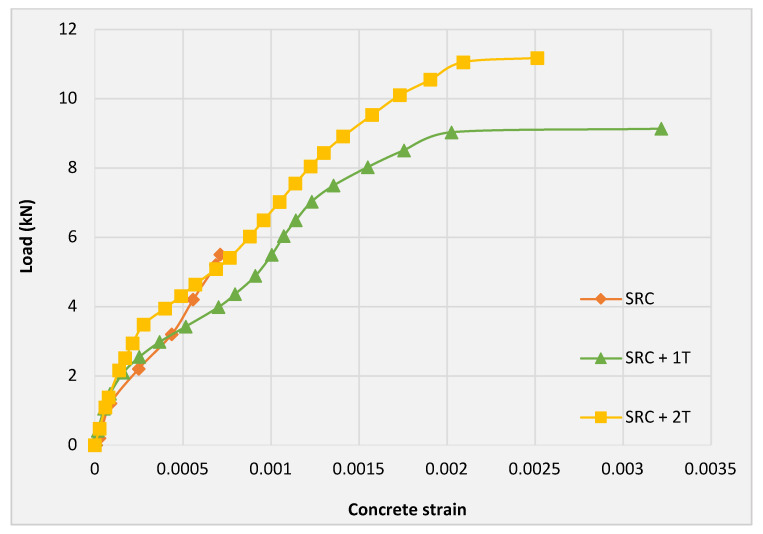
Compressive load versus strain curve of concrete for the three tested slab specimens.

Figure 9 shows the load–strain curves for the concrete in the compression zone for the three tested slab specimens. It can be observed that the concrete compressive strain values are analogous for the three tested slabs until 1.2 kN. Slab SRC + 2T shows lower concrete strain as compared to SRC + 1T at each corresponding load. This can be attributed to the effective bridging mechanism achieved by the top fabric layer of SRC + 2T as compared with the SRC + 1T, in which the stresses were directly transferred from the steel reinforcement to the concrete. The SRC + 1T curve is terminated by a high concrete strain increment, as shown in Figure 9, indicating the rupture of the steel reinforcement after reaching its critical strain. A more or less similar behavior of the load strain curve for textile-reinforced concrete was reported by Papantoniou et al. [17].

### 3.4. Moment–Curvature

**Figure 10:**
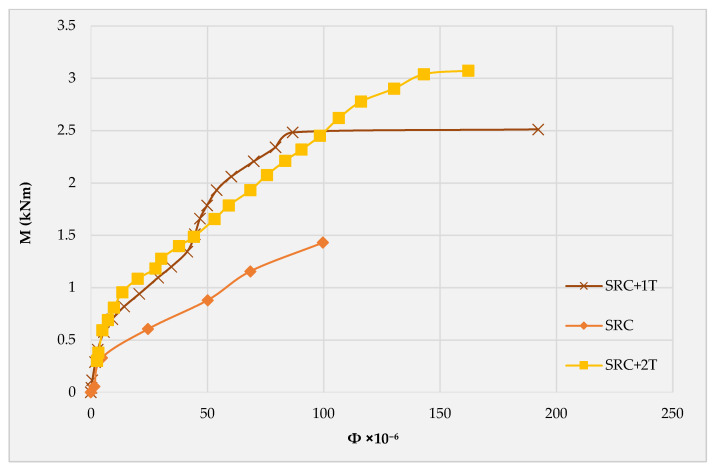
Bending moment versus curvature curve plot for the three tested slab specimens.

Figure 10 shows the experimental moment–curvature curves for the three tested slab specimens. The three curves were generated based on experimental data obtained from the four-point bending test. It can be observed that the three tested slabs show similar curvature until 0.33 kNm, which represents the experimental cracking moment for the SRC slab. This similarity can be attributed to the concrete dominance of the load-deformation behavior during this stage. However, the SRC shows a higher curvature until failure due to the higher concrete compressive strain and the rapid reduction in the depth of the compression zone. While the SRC + 2T depicts a lower curvature until 1.5 kNm, at which the SRC + 1T begins to demonstrate stiffer load-deformation behavior. However, this stiff region is terminated at 2.475 kNm. This can be attributed to the difference in the strain of the steel reinforcement in the two slabs, while the SRC + 1T exhibits higher curvature beyond 2.475 kNm at the same bending moment. This is attributed to the plastic deformation occurrence evident by the high deformation with the slight increase in the load. More or less similar behavior for the moment–curvature of textile-reinforced concrete was reported by Spartali et al. [32].

### 3.5. Deflection Profile

**Figure 11:**
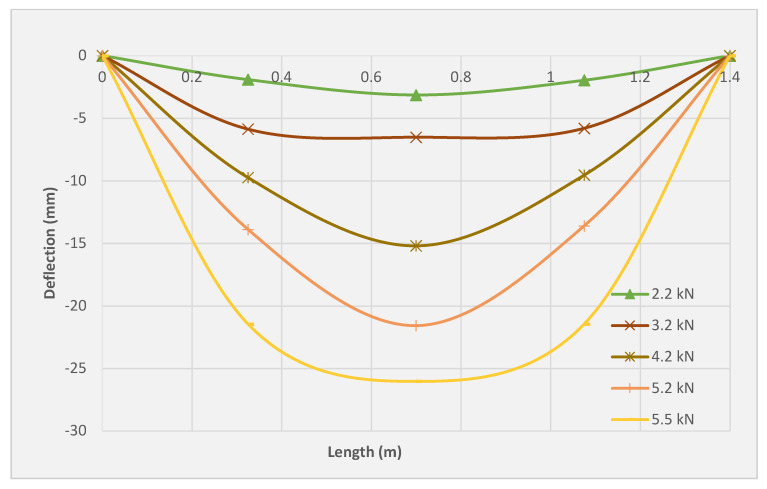
Deflection profile for the SRC tested slab.

**Figure 12:**
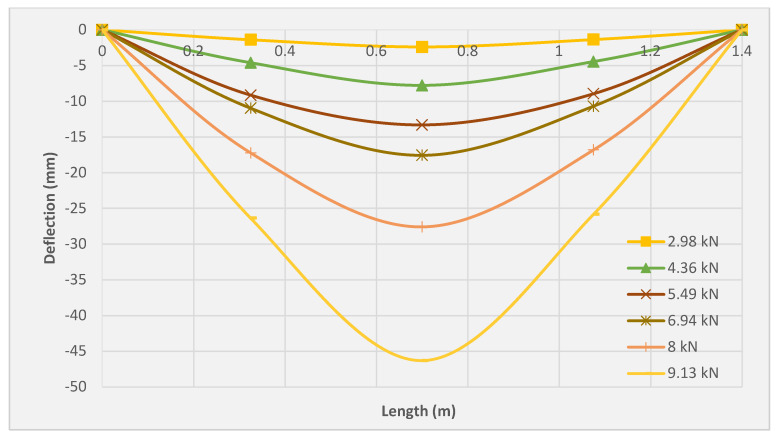
Deflection profile for the SRC + 1T tested slab.

**Figure 13:**
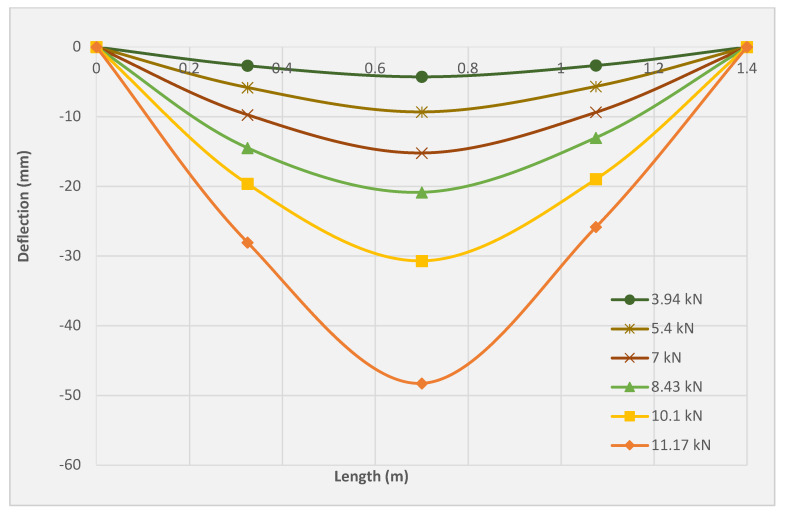
Deflection profile for the SRC + 2T tested slab.

Figure 11, Figure 12 and Figure 13 illustrate the deflection profile of the three tested slabs. The rotational capacity of the three tested slabs can be identified from the deflection profile since the rotation at any point is represented by the ratio of corresponding displacement at the point and its distance from the support. It can be observed that the slabs SRC + 1T and SRC + 2T yielded higher ultimate rotation as compared with the SRC slab. However, SRC exhibited higher rotation compared to the slabs SRC + 1T and SRC + 2T at the same load. The SRC rotation was found to be increased drastically beyond the cracking point, resulting in the extreme reduction of the flexural stiffness. The reason for the lack of crack bridging mechanism is related to the limited redistribution of stresses between the steel reinforcement and the cementitious matrix. Therefore, the crack initiation was limited by the SRC bending zone with fast crack propagation and widening. However, both the SRC + 1T and SRC + 2T slab specimens showed lesser rotation as compared to SRC at the same load due to the sequential redistribution of the stresses between the textile reinforcement and the matrix, which led to the higher tension stiffening, clearly observed during the multiple crack formation. The stress redistribution process between the PAN grid and the concrete matrix delayed the main ascending crack and ensured a slow upward movement of the neutral axis. In slabs SRC + 1T and SRC + 2T, the deflection profiles exhibited an obvious jump at the final stage, especially beyond the yield of the steel reinforcement. This can be attributed to the higher deformation, which the steel reinforcement transmits to the plastic region under a small load. More or less similar deflection profile of textile-reinforced concrete was reported by Volkova et al. [33] and Portal et al. [34].

### 3.6. Ductility Calculation

The load–deformation relationship at the post-elastic stage varies from the elastic stage of loading for the conventional reinforced concrete and the textile-reinforced concrete. Ductility describes the member’s ability to bear the deformation before the failure. The following traditional methods were used to estimate the ductility. The previous section discussed the load–deflection curve, moment–curvature relation, and the rotation of the three tested slab specimens. The yielding of the steel reinforcement in SRC + 2T occurred at double load compared to SRC, while SRC + 1T exhibited the highest yielding load. Therefore, the ductility determination based on the criterion of steel reinforcement yielding is considered unreasonable since the deflection and rotation is increased at this point.

#### 3.6.1. Conventional Method

The yield of the steel reinforcement is considered as a crucial criterion for determining the ductility of the RC members. The ductility is represented by different parameters such as displacement, deformation, rotation, and curvature [42,43]. The following relations show the moment, rotational, and displacement ductility ($\mu_{\varphi }$,$\mu_{\theta },{\;\mathrm{and}\;\mu }_{\Delta })$ calculation based on the curvature and rotation parameters:

(1) $$\mu \varphi =\frac{\varphi_{u}}{\varphi_{y}}$$

(2) $$\mu_{\theta }=\frac{\theta_{u}}{\theta_{y}}$$

(3) $$\mu_{\Delta }=\frac{\Delta_{u}}{\Delta_{y}}$$

In Equations (1)–(3), $\varphi_{u}$ is the ultimate curvature of the element, $\varphi_{y}$ is the yield limit curvature, $\theta_{u}$ is the ultimate plastic hinge rotation, $\theta_{y}$ is the yield of plastic hinge rotation, $\Delta_{u}$ is the ultimate deformation, and $\Delta_{y}$ is the deformation of the steel yielding.

#### 3.6.2. Namaan and Jeong Energy Method

The method developed by Namaan and Jeong [4] suggests the following Equation (4) to determine the ductility:

(4) $$\varphi_{d}=\frac{1}{2}\left(\frac{E_{t}}{E_{e}}+1\right)$$

where E_t_: total absorbed energy, which represents the area under the load–deformation curve; E_e_: elastic energy

The above equation was developed for the hybrid steel and FRP reinforced concrete members. The ductility index values obtained from Equation (4) were found to be equivalent to that computed using Equation (3) for the ideal elastic-plastic behavior. Generally, the materials that exhibit linear-elastic behavior do not have a clear yield point; hence, the ∆_y_is equal to ∆_u_, and the ductility value obtained from Equations (3) and (4) equals unity. However, the accuracy of the obtained results requires the exact determination of the elastic energy portion. The elastic energy can be estimated from the load–deflection curve proposed by [4] by calculating the triangular area, as shown in Figure 1. The slope of the inclined line of the triangular area can be calculated from the following relation given in Equation (5):

(5) $$S\;=\frac{P_{1}S_{1}+\left(P_{2}-P_{1}\right)S_{2}}{P_{2}}$$

where, P_1_ and P_2_ are loads, S_1_ and S_2_ are the identical slopes. The height of the triangle represents the value of the ultimate load, while the base is calculated by dividing the height by the slope value. However, the application of this equation is not limited by a precondition to the reinforcement materials, whatever the mode of failure [4].

### 3.7. Deformability Index

There are two proposed methods to estimate the ductility for the FRP flexural strengthened members as follows:

#### 3.7.1. Deformability of FRP-Strengthened Elements

The deformability index term is suggested by Tan et al. [1] to estimate the ductility of the FRP-strengthened elements. The deformability index can be calculated as follows:

(6) $$\emptyset_{d}=\frac{\Delta_{0.95}}{\Delta_{s}}$$

In Equation (6), $\Delta_{0.95}$ is the deformation (deflection, curvature, rotation, or compressive strains) at 95% of the peak load, and $\Delta_{s}$ is the deformation at 67% of the ultimate load.

#### 3.7.2. Deformability Factor

A deformability index developed by Jaeger et al. [20] can also be used to calculate the ductility of the beams that are reinforced by FRP. This method assumes the 0.001 compressive strain of concrete as a separation limit between the elastic and plastic regions. This limit is considered to correspond to the structural yielding point that occurs in the case of steel reinforcement. This index is known by the J-factor, which represents the product of both the moment and the curvature factors. The moment factor is the ratio of the ultimate moment M_u_ to the moment at 0.001 compressive strain of concrete, while the ratio of the ultimate curvature to the curvature at the 0.001 compressive strain of concrete represents the curvature factor.

The Canadian Standards Association [42] developed a method based on Jaejer et al. [20] to estimate the ductility performance of the concrete members reinforced by brittle reinforced materials. However, this method accounts for both strength and deflection factors; the strength factor represents the ratio of the ultimate load to the load at 0.001 concrete compressive strains. The deflection factor is the ratio of the ultimate mid-span deflection to member mid-span deflection at this concrete strain. In any case, this performance factor must be greater than four for rectangular sections and greater than six for T-sections. The deformability factor is calculated as the product of the strength and deflection factor, as given in Equation (7).

(7) $$\emptyset =\left(\frac{P_{u}}{P_{0.001}}\right)\left(\frac{\Delta_{u}}{\Delta_{0.001}}\right)$$

Table 8 shows the ductility values of the three tested slabs based on the four discussed methods. The conventional ductility method (Method 1) calculated two values of ductility for each of the three tested slabs. The first value was calculated based on the displacement ratio, while the second value was calculated based on the curvature ratio. In the case of SRC and SRC + 2T, the curvature ductility ratio was found to deliver a reduced ductility as compared with the ductility values obtained from the displacement ratio.

In contrast, SRC + 1T show a perfect increase in ductility value based on the curvature ductility ratio in comparison with the displacement ratio. These conflicts are attributed to the immediate increase in the SRC + 1T curvature beyond the yield of steel reinforcement at a slight load increment. However, this highly rapid deformation is caused by the abrupt transformation of tensile stresses from the ruptured PAN fabric to the steel reinforcement. In contrast, the upper carbon fabric layer in SRC + 2T inhibits this immediate increase in deformation after the yielding. Generally, the curvature ductility ratio depends on the compressive strain of the concrete and the tensile strain of the steel reinforcement at yield and at ultimate load, while the displacement ratio depends on the deflection at yield and at ultimate load. However, the displacement ratio results coincide with the findings of Papanicolaou and Papantoniou [15] and Yin et al. [18], wherein an obvious reduction in the ductility for the composite reinforced beam specimens was reported based on Method 1. The higher post cracking stiffness and the significant tension stiffening led to the reduction in deflection, rotation, and curvature values at the yielding of the steel reinforcement in the SRC + 2T slab.

Therefore, based on Method 1, the ductility value was found to be higher for the SRC + 2T specimen due to the presence of two textile layers. Generally, the ductility of such structural members is not related to the yield of the steel reinforcement since the yielding occurrence is delayed to the higher load, which reflects the contribution of the other reinforcement material in bearing considerable stresses. The energy-based method (Method 2) showed a similar ductility value for the SRC as compared to the displacement ratio value, while the ductility values for the SRC + 1T and SRC + 2T slabs tested according to Method 2 showed significant improvement, which is a result of a combination of the textile layers and the steel reinforcement. The SRC + 1T showed higher ductility as compared with the other two slabs since the last showed higher concrete compressive strain and excellent curvature. The textile reinforcement is brittle in nature, i.e., it does not yield plastic deformation, but it has high strain capacity, which improves the ductility of the specimen. The deformability index method (Method 3) showed no ductility improvement for the two tested slabs, SRC + 1T and SRC + 2T. The last method (Method 4) showed no deformability factor value for the SRC since the concrete failed before reaching a compressive strain = 0.001, while in the other two slabs, SRC + 1T and SRC + 2T, the value was found to be greater than four, thereby confirming the mode of failure as ductile.

The obtained results from the four methods indicate a good compatibility between the energy-based method (Method 2) and the deformability factor method (Method 4). The ductility values of slabs SRC + 1T and SRC + 2T, calculated using Method 2, were found to be equal to 60% of that calculated using the deformability method (Method 4). This agrees with the findings of Tan et al. [1], who concluded that the ductility and deformability converge at the ductile failure, while the mode of failure tends to be brittle with an increasing deformability-to-ductility ratio. However, the deformability factor is used to estimate the ductility performance, not the ductility, since it is restricted by the limitation (value > 4) for rectangular sections and (value > 6) for T-sections [28].

**Figure 14:**
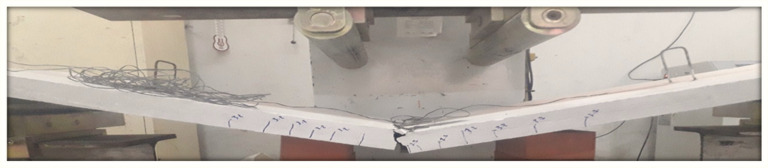
Failure of SRC tested slab.

**Figure 15:**
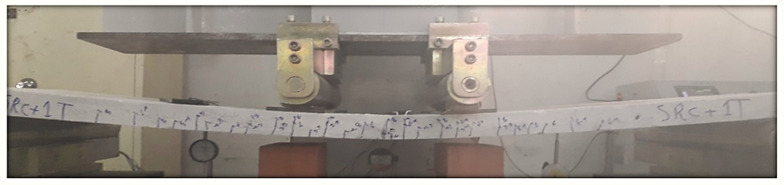
Failure of SRC + 1T tested slab.

**Figure 16:**
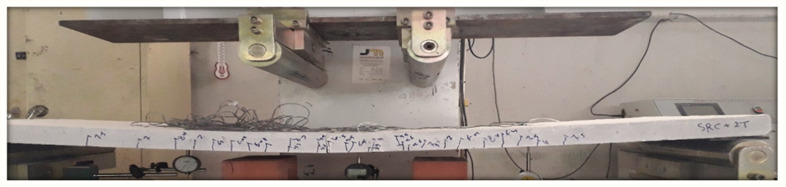
Failure of SRC + 2T tested slab.

The energy-based method is essentially related to the exact determination of the elastic energy portion. However, this determination is related to the average slope of the load–deflection curve. This method has accounted for the effect of both reinforcement types (steel and textile) on the load–deflection behavior of the slab specimens. From the above discussion, the energy-based method can be considered a suitable method of calculating the ductility for such composite reinforced members. However, the ductility values of the slabs SRC + 1T and SRC + 2T computed using the energy method (Method 2) showed good agreement with those in the work of Tan et al. [1], wherein it was concluded that the ductility index for FRP-strengthened flexural members should not decrease to below 2 as a minimum value and 2.5 as a desirable value or greater. In the SRC slab, the ductility value was less than 2.5, indicating a brittle failure (see Figure 14), whereas in the slabs SRC + 1T and SRC + 2T, this value was greater than 2.5 and hence underwent a ductile failure, as observed in the experiments (see Figure 15 and Figure 16). Additionally, a good compatibility of results was observed for the ductility calculated using Method 4, where the values were found to be greater than 4 for slabs SRC + 1T and SRC + 2T, hence satisfying the ductile failure criteria of [43].

## 4. Conclusions

- The yielding point of steel reinforcement should not be considered as a criterion to indicate the ductility of the composite reinforced concrete flexural members since the second reinforcement type may delay the yielding of the steel reinforcement.
- The ductility calculation based on displacement and curvature ratios exhibits clear conflicts since the former depends on the deflection at the yield and ultimate loads, while the curvature ratio is affected by the compressive strain of the concrete and the tensile strain of the steel reinforcement at yield and at ultimate load.
- The method of calculating the ductility of the composite reinforced concrete flexural members using the ratio of the ultimate deflection to the deflection of the service load yields an arbitrary value.
- The deformability method, which was developed by the Canadian Standards Association (2012) (CAN/CSA S-806 12), showed a rational ductility value for the two (SRC + 1T and SRC + 2T) tested slabs.
- In the SRC slab, the ductility value was found to be equivalent for both displacement ratio and energy methods, while in the case of the slabs SRC + 1T and SRC + 2T, the ductility values calculated using the displacement method was found to be equal to 60% of that calculated using the energy method. However, the last method proposed the value of 4 as a limitation that separates the brittle and ductile failures for the flexural reinforced rectangular members.
- The energy-based method was found to be the most suitable method of calculating the ductility for the composite reinforced concrete flexural members since (1) it considers a mutual, cooperative contribution of the textile and steel reinforcements in sustaining the applied stresses, and (2) it considers the variation in the slope of the load–deflection curve.

## Figures and Tables

**Table 1:** Constituents of the fine-grained concrete.

Cement (kg/m^3^)	650
Silica fume (kg/m^3^)	65
Sand (0–0.6) mm (kg/m^3^)	1215
Superplasticizer (kg/m^3^)	15.37
Water (kg/m^3^)	250
Cylinder compressive strength (28) day (MPa)	47.2
Flow ability, diameter in (mm)	250

**Table 2:** The tensile test data of steel reinforcement.

Bar Diameter (mm)	Measured Diameter (mm)	Yield Stress (MPa)	Strain at Yield Stress (Micro Strain)	Ultimate Stress (MPa)	Modulus of Elasticity (MPa)
4	4.45	390	2000	464.7	195,000

**Table 3:** Technical data sheet of PAN textile grid provided by the manufacturer.

Material	PAN Textile
Weight (kg/m^2^)	0.16 ± 0.010
Width (mm)	1000
Thickness (mm)	0.2
Mesh size (mm)	25 × 330
Tensile strength (MPa)	3530
Tensile Modulus (MPa)	230,000
Color	Black

**Table 4:** Reinforcement details for the three slab specimens.

Slab Symbol	A_s_. mm^2^	A_T_ mm^2^	Steel σt MPa	Textile σt MPa	Location of the Reinforcements (From the Bottom)
SRC	77.75	–	390	–	Steel: 10 mm
SRC + 1T		9		3530	Steel: 10 mm Textile layer 1T: 9.9 mm
SRC + 2T		18			Steel: 10 mm Textile layer 1T: 9.9 mm Textile layer 2T: 14.1 mm

**Table 5:** Mechanical properties of concrete.

Type of Concrete Mix	Compressive Strength (MPa)	Tensile Strength (MPa)	Flexural Strength (MPa)	Modulus of Elasticity (GPa)
Concrete (TRC)	45–51	4.1–44.44	6.05–6.3	31.528

**Table 6:** Strain gauge properties used in the present study.

Gauge Type	Resistance in Ω	Grid Size (mm)	Gauge Dimensions	
			Length (mm)	Width (mm)
BF350-1AA	120	1 × 3.75	4.7	4.6
BF120-20AA	120	20 × 3.5	25	5

**Table 7:** The test results of the three tested slabs.

Slab Symbol	Cracking Load (kN)	Yield Load (kN)	Ultimate Load (kN)	Mid-Span Deflection at Yield (mm)	Mid-Span Deflection at Ultimate Load (mm)
SRC	1.2	4.2	5.5	15.2	26.04
SRC + 1T	2.09	9.03	9.13	33.46	46.3
SRC + 2T	3.48	8.045	11.17	19.17	48.28

**Table 8:** Comparison of ductility values for the three tested slabs computed using different methods.

Slab Symbol	Method (1)		Method (2)	Method (3)	Method (4)
	$\frac{\Delta_{u}}{\Delta_{y}}$	$\frac{\varphi_{u}}{\varphi_{y}}$	$\frac{1}{2}\left(\frac{E_{t}}{E_{e}}+1\right)$	$\frac{\Delta_{0.95}}{\Delta_{s}}$	$\left(\frac{P_{u}}{P_{0.001}}\right)\left(\frac{\Delta_{u}}{\Delta_{0.001}}\right)$
SRC	1.71	1.46	1.71	2.05	–
SRC + 1T	1.17	2.22	3.37	2.11	5.78
SRC + 2T	2.51	1.94	3.06	2.18	5.04

## Data Availability

The data used to support the findings of this study are included in the article.

## Sample Availability

Not available.
